# C‐reactive protein flare‐response predicts long‐term efficacy to first‐line anti‐PD‐1‐based combination therapy in metastatic renal cell carcinoma

**DOI:** 10.1002/cti2.1358

**Published:** 2021-12-06

**Authors:** Niklas Klümper, Philipp Schmucker, Oliver Hahn, Benedikt Höh, Angelika Mattigk, Severine Banek, Jörg Ellinger, Julia Heinzelbecker, Danijel Sikic, Markus Eckstein, Arne Strauß, Friedemann Zengerling, Michael Hölzel, Philip Zeuschner, Charis Kalogirou

**Affiliations:** ^1^ Department of Urology and Paediatric Urology University Hospital Bonn (UKB) Bonn Germany; ^2^ Institute of Experimental Oncology University Hospital Bonn (UKB) Bonn Germany; ^3^ Department of Urology and Paediatric Urology Julius Maximilians University Medical Center of Würzburg Würzburg Germany; ^4^ Department of Urology University Medical Center Göttingen Göttingen Germany; ^5^ Department of Urology University Hospital Frankfurt Goethe University Frankfurt am Main Frankfurt Germany; ^6^ Department of Urology and Paediatric Urology University Hospital Ulm Ulm Germany; ^7^ Department of Urology and Paediatric Urology Saarland University Homburg/Saar Germany; ^8^ Department of Urology and Pediatric Urology University Hospital Erlangen Friedrich‐Alexander‐University Erlangen‐Nuremberg Erlangen Germany; ^9^ Comprehensive Cancer Center Erlangen‐EMN (CCC ER‐EMN) Erlangen Germany; ^10^ Institute of Pathology University Hospital Erlangen Friedrich‐Alexander‐University Erlangen‐Nuremberg Erlangen Germany

**Keywords:** biomarker, checkpoint inhibition, C‐reactive protein, CRP flare‐response, immunotherapy, metastatic renal cell carcinoma

## Abstract

**Objectives:**

Immune checkpoint blockade (IO) has revolutionised the treatment of metastatic renal cell carcinoma (mRCC). Early C‐reactive protein (CRP) kinetics, especially the recently introduced CRP flare‐response phenomenon, has shown promising results to predict IO efficacy in mRCC, but has only been studied in second line or later. Here, we aimed to validate the predictive value of early CRP kinetics for 1st‐line treatment of mRCC with αPD‐1 plus either αCTLA‐4 (IO+IO) or tyrosine kinase inhibitor (IO+TKI).

**Methods:**

In this multicentre retrospective study, we investigated the predictive potential of early CRP kinetics during 1st‐line IO therapy. Ninety‐five patients with mRCC from six tertiary referral centres with either IO+IO (*N* = 59) or IO+TKI (*N* = 36) were included. Patients were classified as CRP flare‐responders, CRP responders or non‐CRP responders as previously described, and their oncological outcome was compared.

**Results:**

Our data validate the predictive potential of early CRP kinetics in 1st‐line immunotherapy in mRCC. CRP responders, especially CRP flare‐responders, had significantly prolonged progression‐free survival (PFS) compared with non‐CRP responders (median PFS: CRP flare‐responder: 19.2 months vs. responders: 16.2 vs. non‐CRP responders: 5.6, *P* < 0.001). In both the IO+IO and IO+TKI subgroups, early CRP kinetics remained significantly associated with improved PFS. CRP flare‐response was also associated with long‐term response ≥ 12 months.

**Conclusions:**

Early CRP kinetics appears to be a low‐cost and easy‐to‐implement on‐treatment biomarker to predict response to 1st‐line IO combination therapy. It has potential to optimise therapy monitoring and might represent a new standard of care biomarker for immunotherapy in mRCC.

## Graphical abstract

In this multicentre study, we investigated the predictive potential of early serum C‐reactive protein (CRP) kinetics during 1st‐line immunotherapy in metastatic renal cell carcinoma (mRCC). Out of 95 patients with mRCC, those with significant CRP flare‐response or decrease in CRP levels had superior outcomes. This potential biomarker could improve therapy monitoring of mRCC and eventually also treatment stratification.

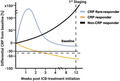

## Introduction

First‐line treatment of metastatic renal cell carcinoma (mRCC) has changed substantially in recent years because of the introduction of a new therapy regimen, mainly based on immune checkpoint inhibition (IO).^1^, ^2^, ^3^ Currently, two different types of approved first‐line combination therapies are applied equivalently for the treatment of intermediate and poor‐risk metastatic mRCC according to IMDC (International Metastatic Renal Cell Carcinoma Database Consortium Score): (1) a combination of αPD‐1 and αCTLA‐4 immune checkpoint inhibitors as well as (2) a combination of αPD‐1 (or αPD‐L1) with small‐molecule tyrosine kinase inhibitors (TKI) targeting the vascular endothelial growth factor receptor (VEGFR).^4^, ^5^, ^6^, ^7^, ^8^ In essence, these two regimens can be classified as an intensified immune checkpoint inhibition (IO+IO) and a combination of immune checkpoint inhibition plus anti‐angiogenic therapy (IO+TKI).

However, only a subset of patients responds to these first‐line IO combination therapies. On the one hand, reliable predictive biomarkers could identify early therapy failure, which is of high clinical relevance. On the other hand, severe unnecessary side effects could be avoided, and the individual therapy regimen could be further optimised.

In general, IO treatment success is based on the induction of an antitumor immune response. C‐reactive protein (CRP) is a serum acute‐phase reactant and clinically widely used surrogate biomarker for the assessment of systemic inflammation. The occurrence and kinetics of systemic inflammatory response reflected by serum CRP has been implicated with clinical outcome and treatment response in diverse cancer entities, including urothelial cancer, non‐small‐cell lung cancer and mRCC.^9^, ^10^, ^11^, ^12^, ^13^, ^14^ Several studies investigated CRP levels at initial diagnosis or baseline before therapy initiation and associated increased systemic inflammation with poor oncologic prognosis. As cancers can also induce chronic inflammation, on‐treatment CRP kinetics may have predictive value for immunotherapy treatment success.^15^, ^16^

Just recently, Fukuda *et al*. described the CRP ‘flare‐response’ phenomenon defined by an early CRP increase after IO treatment initiation with a subsequent drop below baseline. These early CRP changes appear to mirror the dynamic phase of systemic inflammation after inducing the desired antitumoral immune response on IO therapy.^15^ Of note, this novel concept allowed an accurate prediction of therapy success in 42 mRCC patients treated with αPD‐1. However, the investigated cohort only included a limited patient number and αPD‐1 monotherapy was administered as 2nd‐line (or later) post‐TKI treatment. As IO monotherapy will occur less frequently in the future, our study aimed to investigate the emerging phenomenon of CRP flare‐response in a multicentre mRCC cohort receiving either IO+IO or IO+TKI as 1st‐line standard of care therapy.

## Results

### Patient characteristics

Between November 2017 and April 2021, 95 were included in this study (for comprehensive patient characteristics, see Table 1). In brief, *N* = 59 patients (62.5%) received IO+IO and *N* = 36 (37.5%) IO+TKI. The median patient age was 67 (interquartile range, IQR 57.5–75.0) years, and 64 (67.4%) patients were male. Most patients had been diagnosed with clear cell RCC (71.6%), had an Eastern Co‐operative of Oncology Group (ECOG) score ≤ 1 (91.6%) and were IMDC intermediate risk (65.3%). The median follow‐up was 11.1 (5.6–17.3) months.

**Table 1:** Comparison of baseline patient and tumor demographics between CRP flare‐responders, CRP responders and non‐CRP responders

	Total cohort	Early CRP kinetics			*P‐*value
		Non‐CRP responder	CRP responder	CRP flare‐responder	
No. of patients	95	48 (50.1%)	34 (35.8%)	13 (13.7%)	
Age	67.0 (57.50–75.0)	67.5 (54.8–77.0)	68.0 (58.3–72.8)	67.0 (64.0–72.0)	0.987
Male gender	64 (67.4%)	32 (66.7%)	23 (67.6%)	9 (69.2%)	1
ECOG					
0	42 (44.2%)	19 (39.6%)	17 (50.0%)	6 (46.2%)	0.886
1	45 (47.4%)	24 (50.0%)	14 (41.2%)	7 (53.8%)	
2	6 (6.3%)	4 (8.3%)	2 (5.9%)	0 (0%)	
3	1 (1.1%)	1 (2.1%)	0 (0%)	0 (0%)	
IMDC					
Favorable	16 (16.8%)	9 (18.8%)	3 (8.8%)	4 (30.8%)	0.352
Intermediate	62 (65.3%)	31 (64.6%)	23 (67.6%)	8 (61.5%)	
Poor	16 (16.8%)	7 (14.6%)	8 (23.5%)	1 (7.7%)	
Synchronous metastasis	65 (68.4%)	33 (68.8%)	24 (70.6%)	8 (61.5%)	0.805
Prior nephrectomy	75 (78.9%)	39 (81.3%)	25 (73.5%)	11 (84.6%)	0.668
Clear cell histology	68 (71.6%)	35 (72.9%)	23 (67.6%)	10 (76.9%)	1
Tumor stage					
T1	32 (33.7%)	16 (33.3%)	12 (35.3%)	4 (30.8%)	0.916
T2	8 (8.4%)	4 (8.3%)	3 (8.8%)	1 (7.7%)	
T3	33 (34.7%)	18 (37.5%)	9 (26.5%)	6 (46.2%)	
T4	7 (7.4%)	5 (10.4%)	1 (2.9%)	1 (7.7%)	
Lymph nodes					
N0	44 (46.3%)	22 (45.8%)	16 (47.1%)	6 (46.2%)	0.834
N1	21 (22.1%)	10 (20.8%)	9 (26.5%)	2 (15.4%)	
NX	22 (23.2%)	12 (25.0%)	6 (17.6%)	4 (30.8%)	
Grade					
1	4 (4.2%)	3 (6.3%)	0 (0%)	1 (7.7%)	0.063
2	27 (28.4%)	18 (37.5%)	4 (11.8%)	5 (38.5%)	
3	32 (33.7%)	16 (33.3%)	13 (38.2%)	3 (23.1%)	
4	14 (14.7%)	4 (8.3%)	7 (20.6%)	3 (23.3%)	
Positive margins	6 (6.3%)	3 (6.3%)	2 (5.9%)	1 (7.7%)	0.668
Radiotherapy	29 (30.5%)	15 (31.3%)	11 (32.4%)	3 (23.1%)	0.881
Time to therapy	7.0 (1.0–25.5)	10.0 (2.0–32.3)	3.0 (1.0–10.8)	10.0 (4.0–33.0)	**0.025**
1st‐line therapy					
IO+IO	59 (62.1%)	32 (66.7%)	22 (64.7%)	5 (38.5%)	0.185
IO+TKI	36 (37.9%)	16 (33.3%)	12 (35.3%)	8 (61.5%)	
Baseline CRP in mg dL^−1^	2.21 (0.50–14.86)	0.87 (0.23–2.35)	8.87 (3.94–58.42)	1.73 (0.78–9.17)	**< 0.001**
No. of CRP measurements first 3 months	5.0 (3.0–8.0)	5.0 (3.0–8.0)	4.0 (3.0–7.75)	7.0 (5.0–8.0)	0.232

Significant *P*‐values are displayed in bold.

### Response and outcomes by early CRP kinetics

**Figure 1:**
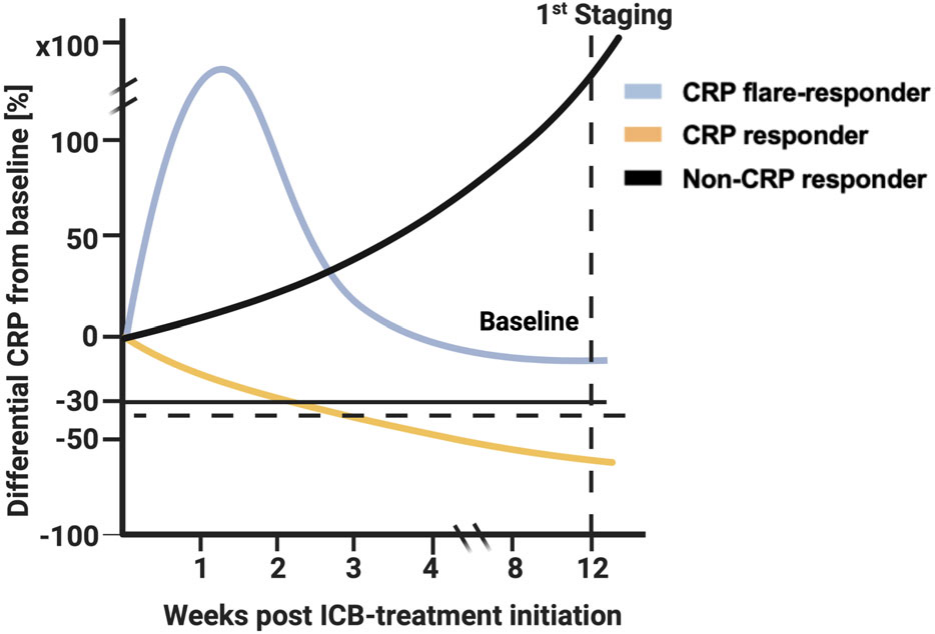
Model of early C‐reactive protein (CRP) kinetics with the CRP flare‐response phenomenon, CRP response and non‐CRP response after IO therapy initiation up to 1st staging. Adapted from Fukuda *et al*.,^15^ created with BioRender.com.

**Figure 2:**
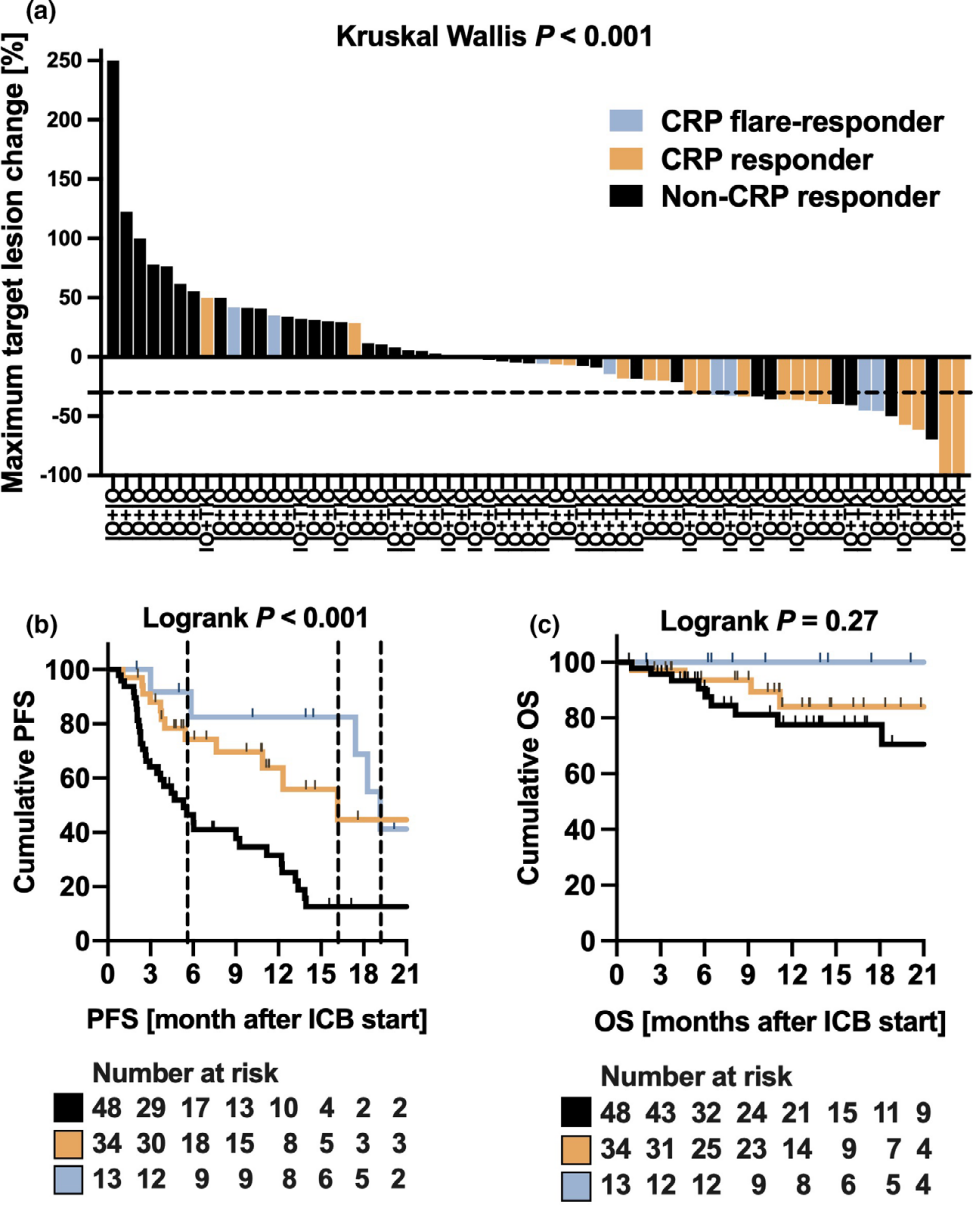
**(a)** Waterfall plot depicting the maximum target lesion change in the three defined CRP kinetic subgroups of the multicentre mRCC cohort (*N* = 64; no RECIST data available for *N* = 31 patients). **(b, c)** Progression‐free (PFS) and overall survival (OS) after IO treatment initiation for CRP flare‐responder (*N* = 13), CRP responder (*N* = 34) or non‐CRP responder (*N* = 48). Median PFS is depicted as a dotted line, median OS not reached.

Thirteen (13.7%) patients were classified as CRP flare‐responders, 34 (35.8%) as CRP responders and 48 (50.5%) as non‐CRP responders (Figure 1). There were no significant differences in baseline characteristics (Table 1), except median time from initial diagnosis to start of systemic therapy and median baseline CRP values, as CRP non‐responders had significantly lower CRP values than CRP (flare) responders (*P* < 0.001). The median follow‐up length did not differ between CRP dynamic groups (*P* = 0.292). A median of 6.0 doses (4.0–14.5) of intravenous IO therapy was administered in the whole study population and the amount differed significantly between the three subgroups (*P* = 0.016). CRP flare‐responders, CRP responders and non‐CRP responders had a median maximum target lesion change of −16.3% (IQR −32.5% to −1.0%), −31.7% (IQR −38.7% to −12.5%) and 6.8% (IQR −7.8% to 40.8%), correspondingly (Figure 2a, *P* < 0.001). Five (5/12 = 41.7%) patients in the CRP flare‐responder, 14 (14/31 = 45.2%) in the CRP responder and 8 (8/48 = 17.0%) in the non‐CRP responder group had an objective therapy response, which differed significantly (*P* = 0.019).

### Survival analysis by early CRP kinetics

**Figure 3:**
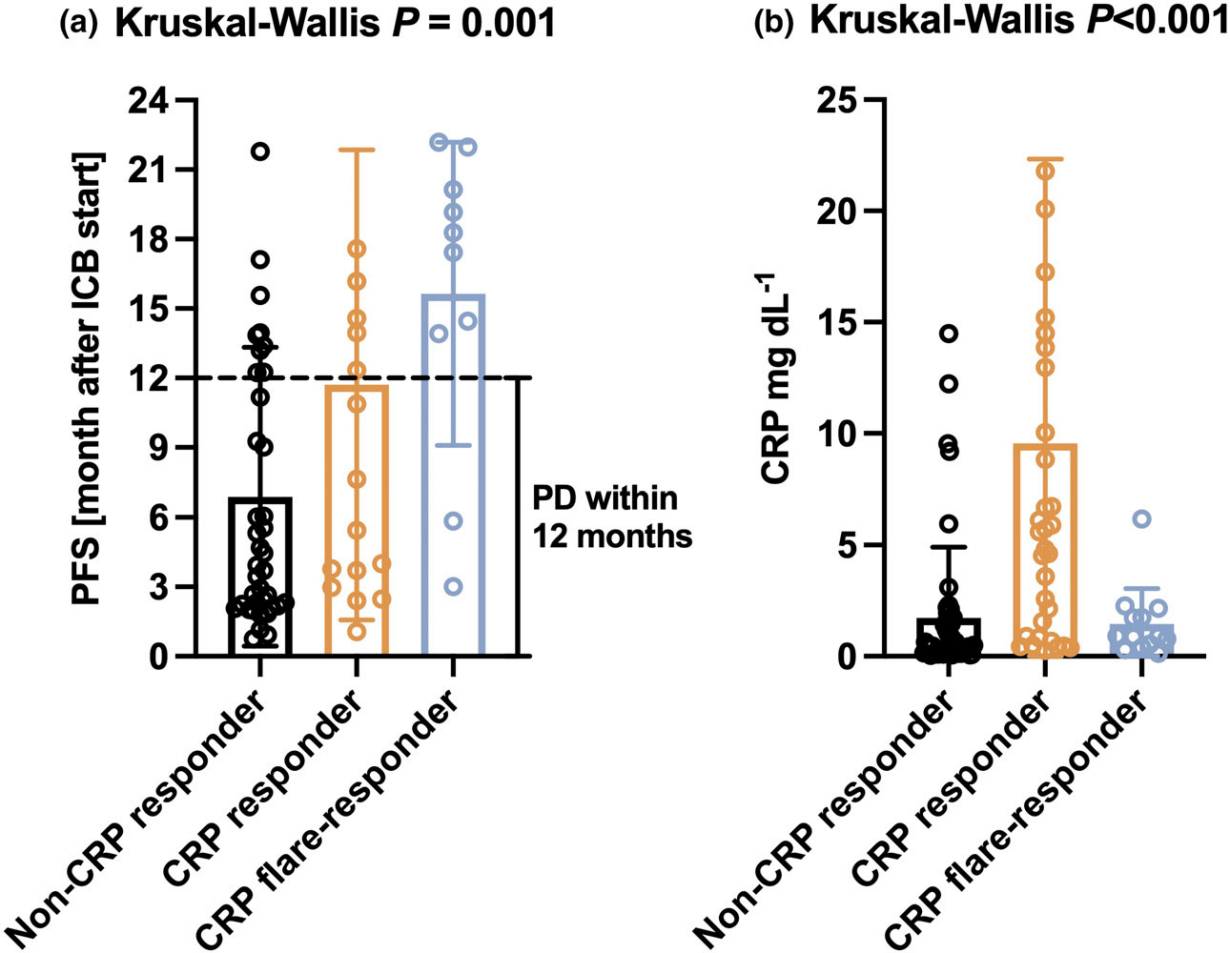
**(a)** Duration of immunotherapy depending on the CRP dynamic subgroups is shown. Long‐term IO response was defined as ≥ 12 months. Patients with ongoing IO therapy but follow‐up less than 12 months were excluded for this analysis because achievement of long‐term response cannot be stratified. **(b)** Boxplot depicting baseline CRP serum concentration stratified by CRP dynamic groups (mean with SD).

The median progression‐free survival (PFS) after initiation of IO treatment was 5.6 months (95% CI 3.4–12.2 months) for non‐CRP responders, 16.2 months for CRP responders (95% CI 10.9 months – not reached) and 19.2 months for CRP flare‐responders (95% CI 17.4 months – not reached) and differed significantly (Figure 2b). When the overall cohort was divided into subgroups of patients receiving IO+IO or IO+TKI, early CRP kinetics remained significantly associated with PFS on immunotherapy in both groups (Supplementary figure 1). Of note, the majority of CRP flare‐responders (8/10 = 80.0%) showed long‐term therapy response lasting ≥ 12 months. Thus, the mean duration of IO response differed significantly between the groups (*P* = 0.001; Figure 3a).

### Cox regression

In the univariate Cox regression, early CRP dynamics was the only factor that was significantly associated with the PFS, besides ECOG score (Table 2). Of note, baseline CRP level was not associated with PFS, but highest in the CRP response group (Figure 3b, *P* < 0.001). Compared to Non‐CRP responders, CRP responders had a risk reduction for progression of 68% [hazard ratio HR 0.32, 95% confidence interval (CI) 0.17–0.62, *P* = 0.001] and CRP flare‐responders of 73% (HR 0.27 95% CI 0.11–0.66, *P* = 0.004). No other patient or tumor‐related factor had an impact on the PFS after IO treatment initiation. In the multivariate Cox regression model, the impact of CRP dynamics and ECOG score remained significant (Table 2). Additionally, the therapy regimen, baseline CRP (HR 1.01, *P* = 0.021) and T stadium (T2 vs. T1: OR 7.56, *P* = 0.004) now also had a significant impact on progression.

**Table 2:** Uni‐ and multivariable Cox regression analyses for progression‐free survival

	Univariable		Multivariate	
	HR (95% CI)	*P*‐value	HR (95% CI)	*P*‐value
Therapy				
IO+IO	ref.	0.059	ref.	0.030
IO+TKI	0.58 (0.33; 1.02)		0.29 (0.1; 0.89)	
CRP dynamics				
No response	ref.	< 0.001	ref.	0.002
Responder	0.32 (0.17–0.62)	0.001	0.22 (0.06; 0.76)	0.017
Flare‐responder	0.27 (0.11–0.66)	0.004	0.19 (0.06; 0.60)	0.005
Baseline CRP	1.00 (0.97; 1.04)	0.893	1.01 (1.00; 1.02)	0.021
Age	0.98 (0.96; 1.00)	0.094	0.98 (0.95; 1.01)	0.246
Gender				
Male	ref.	0.545	ref.	0.987
Female	1.19 (0.68; 2.10)		0.63 (0.28; 1.43)	
ECOG				
0	ref.	0.010	ref.	0.001
1	1.74 (0.98–3.09)	0.058	3.40 (1.57; 7.36)	0.002
2	6.46 (2.1–19.90)	0.001	23.60 (4.1; 136.03)	< 0.001
3	0.0	0.980	0.0	0.978
IMDC				
Favorable	ref.	0.620	ref.	0.330
Intermediate	1.40 (0.67; 2.94)	0.372	0.47 (0.13; 1.70)	0.249
Poor	1.51 (0.60; 3.83)	0.382	0.84 (0.17; 4.05)	0.826
Histology				
Clear cell	ref.	0.233	ref.	0.108
Non‐clear cell	1.54 (0.76; 3.11)		2.40 (0.83; 7.00)	
pT stadium				
pT1	ref.	0.500	ref.	0.022
pT2	1.69 (0.65; 4.38)	0.280	7.56 (1.91; 29.87)	0.004
pT3	1.57 (0.81; 3.05)	0.182	3.04 (1.14; 8.08)	0.026
pT4	1.76 (0.58; 5.39)	0.320	5.66 (1.31; 24.47)	0.020

Regarding OS, only ECOG had a significant impact in the univariate Cox regression, as patients with worse performance status had an increased risk for death from any cause (Supplementary table 1). However, this association did not remain significant in the multivariate Cox regression model.

## Discussion

In this retrospective multicentre study, we validate that early CRP kinetics on immunotherapy is a promising predictive biomarker in mRCC. Because of its low cost and wide clinical availability, the CRP kinetic assessment is easy to implement into daily clinical practice and may prove to be a valuable tool for IO therapy monitoring in the future.

In our cohort consisting of 95 patients with either IO+IO‐ or IO+TKI‐based first‐line therapy, CRP flare‐response was associated with long‐term response and improved PFS in the αPD‐1‐based first‐line setting of mRCC. However, in our mRCC cohort, early CRP kinetics showed no significant association with OS, which is most likely attributed to the relatively low number of events in the cohort. Since the new 1st‐line combination therapies in mRCC remarkably prolong OS, we plan to reanalyse this cohort after extending the follow‐up period. Further, early CRP kinetics was significantly associated with improved PFS in both subgroups (IO+IO and IO+TKI), leading us to conclude that early CRP kinetics is a robust predictive biomarker in mRCC independent of the chosen first‐line treatment combination. Since Fukuda *et al*. described the predictive value of early CRP kinetics for nivolumab monotherapy in 2nd line or later, it appears that early CRP kinetics can therefore be used to optimise treatment monitoring for all αPD‐1‐based therapies in mRCC.^15^ We consider this to be a particularly important information for the daily clinical routine, as early CRP kinetics could be used as a simple and cost‐effective biomarker for all immunotherapy regimen in mRCC. Non‐CRP response would lead to earlier staging, and in the event of tumor progression, allow clinicians to administer alternative and more effective therapies while preventing exposure to potentially life‐threatening toxic effects of immunotherapy.^17^ In our analysis, early CRP kinetics appear to have the potential to predict treatment response before initial staging and thus lead to earlier treatment modification, which could ultimately improve the clinical course of mRCC patients.

In addition, it appears to be highly relevant to sensitise clinicians to the characteristic CRP flare‐response phenomenon, as a rapid increase in CRP could be the result of a desirable antitumor immune response. CRP flare‐response should, in the absence of other clinical symptoms, thus not be misinterpreted as a bacterial infection or another side effect after IO therapy initiation especially since antibiotic‐induced dysbiosis can compromise the clinical activity of immunotherapy by modulating, for example the gut microbiome.^18^

Exploring the tumor immunologic basis of the differential CRP kinetics after initiation of immunotherapy might further enhance our understanding of the interplay between the RCC tumor cells and its tumor microenvironment (TME).^19^, ^20^, ^21^ Baseline serum CRP concentration, which may reflect the baseline RCC immunogenicity, differs significantly between the CRP response groups. The low baseline CRP level in flare‐responders could be an indirect surrogate for low or absent chronic inflammation caused by the tumor burden. Thus, we hypothesise that in treatment‐naïve RCC tissue, differential immune phenotypes may predict early CRP kinetics as IO treatment triggers distinct immune cell infiltration patterns to enrich the TME. Thereafter, the induction of an antitumor immune response leads to systemic inflammation through the release of inflammatory mediators, which can ultimately be measured by serum CRP. To address this hypothesis, future studies will need to perform comprehensive phenotyping of treatment‐naïve tumor tissue, followed by integration of the early CRP kinetic subset. From a clinical point of view, the identification of specific TME patterns in treatment‐naïve RCC tissue that robustly predict early CRP kinetics and response would be of high relevance to stratify our patients before therapy, especially since currently available predictive tools such as PD(L)‐1 immunohistochemistry (IHC) only play a minor role in mRCC.^22^ From a cancer‐immunologic point of view, it would be of high relevance to identify the distinct immune signatures associated with non‐CRP response and IO treatment failure to identify potential targets for tailored combination therapy in this immunotherapy‐unresponsive RCC subgroup.

Increased baseline concentration of inflammation markers such as CRP or IL‐8 before oncological treatment has also been associated with worse clinical outcome in mRCC patients treated with immunotherapy elsewhere, but the dynamic and early change in systematic inflammation after therapeutic intervention was mostly neglected.^9^, ^10^, ^12^, ^23^, ^24^ In mRCC, an early decrease in CRP after initiation of TKI therapy has already been associated with improved response and survival.^25^ Only recently, the predictive potential of characteristic longitudinal changes in CRP, especially the newly described flare‐response, during the first 3 months of αPD‐1 monotherapy in the post‐TKI setting has been highlighted. We evaluated the predictive value of early CRP dynamics in a larger, multicentre and more clinically relevant cohort in the first‐line setting in mRCC and demonstrated that CRP responders and particularly CRP flare‐responders showed favorable progression‐free survival (PFS) and mostly durable treatment response. Further studies will have to clarify whether the flare‐response kinetics of systemic inflammation can be sharpened by replacing the relatively nonspecific CRP with other acute‐phase reactants or immune mediators. After prospective validation of the predictive potential of early CRP kinetics in mRCC and possibly in additional tumor subtypes, we propose early CRP kinetics as a promising on‐treatment biomarker for stratifying our patients in the era of immuno‐oncology.

Despite noteworthy strengths, such as the multicentre approach and the comparably large study cohort, our study also has several limitations. First and foremost, we acknowledge that the study is limited by its observational nature and the relatively short follow‐up time, especially for the meaningful endpoint OS. Moreover, our results should be interpreted within the limitations of the retrospective design. CRP was measured in different routine clinical laboratories at the study centres and without a standardised scheme, so some CRP flare‐responses may have been missed. In addition, modification of the new and not prospectively validated early CRP kinetic concept might increase its predictive value. Nevertheless, we propose a prospective evaluation of our results in future studies, based on our promising retrospective data.

If prospectively validated, we propagate that early CRP kinetics should be assessed as an easy‐to‐implement, non‐invasive biomarker during IO combination therapy in mRCC as the new standard of care, as early detection of treatment success and failure might have the potential to optimise treatment monitoring and adjustment and to prevent exposure to potentially life‐threatening side effects of IO therapy.

## Methods

In this retrospective multicentre study, *N* = 118 consecutive mRCC patients from six German tertiary referral centres receiving either first‐line IO+IO (αPD‐1/nivolumab + αCTLA4/ipilimumab) or IO+TKI (α PD‐1/pembrolizumab + VEGFR‐TKI/axitinib) were screened. Patients with CRP measurements at baseline (closest to treatment initiation, maximum 6 weeks before), at least once within the first month of treatment and at least one further CRP at the time of first staging or clinical progression were included in the study. Of the total *N* = 118 patients initially studied, *N* = 23 were excluded due to missing CRP values, resulting in a study cohort of *N* = 95 patients.

This study was conducted according to the Declaration of Helsinki and approved by the responsible ethical review board (reference #20201211‐01).

The patient demographics and baseline parameters including IMDC risk criteria were obtained. Tumor response was graded according to response evaluation criteria in solid tumors (RECIST v1.1).^26^ Therapy outcomes were compared among the three characteristic therapy groups, defined by diverging CRP dynamics. According to the earlier definition by Fukuda *et al*., ‘CRP flare‐responders’ were defined as an early increase in CRP levels to more than double from baseline within 1 month after therapy initiation and a subsequent decrease below the baseline within 3 months. Patients with a decrease by ≥ 30% from baseline within 3 months without flare‐response were classified as ‘CRP responders’, all other patients as ‘non‐CRP responders’ (Figure 1).^15^ To define these CRP dynamic groups, CRP at baseline, during the first month after treatment initiation and follow‐up visits was obtained. Serum CRP concentration was measured in accredited routine laboratories in each participating centre and is given in mg dL^−1^ (clinical reference < 0.5 mg dL^−1^).

Categorical variables were reported as frequencies and proportions, continuous data as the median and range. Fisher's exact tests, Mann–Whitney *U*‐tests and Kruskal–Wallis tests were applied to perform intergroup comparisons. The PFS and OS, including 95% confidence intervals, were estimated from the day of treatment initiation until the respective event using the Kaplan–Meier method and compared with log‐rank tests. Progression was defined according to the RECIST v1.1 criteria including death from any cause. To compare the impact of the therapy regimen (IO+IO vs. IO+TKI), CRP dynamics (CRP flare‐responder, CRP responder vs. non‐CRP responder), baseline patient (age, gender, ECOG) and tumor‐related parameters (e.g. IMDC, histology, pT‐stage) on OS and PFS, univariate and multiple Cox regressions were conducted. Patient age and CRP baseline were defined as continuous, all others as categorical variables. In the event of missing data, cases were excluded from the analysis. Statistical analyses were performed with SPSS version 25 (IBM, Armonk, NY, USA), R (version x64 4.0.3) and GraphPad Prism 9 (GraphPad Software Inc, CA, USA). All statistical tests were two‐sided, and *P*‐values < 0.05 were considered significant.

## Conflict of interest

The authors declare no conflict of interest.

## Author contributions

**Niklas Klümper:** Conceptualization; Formal analysis; Funding acquisition; Investigation; Methodology; Project administration; Resources; Supervision; Visualization; Writing – original draft. **Philipp Schmucker:** Formal analysis; Investigation; Methodology; Project administration; Visualization; Writing – original draft. **Oliver Hahn:** Data curation; Investigation; Writing – review & editing. **Benedikt Höh:** Formal analysis; Investigation; Writing – review & editing. **Angelika Mattigk:** Formal analysis; Investigation; Writing – review & editing. **Severine Banek:** Resources; Supervision; Writing – review & editing. **Jörg Ellinger:** Resources; Supervision; Writing – review & editing. **Julia Heinzelbecker:** Resources; Supervision; Writing – review & editing. **Danijel Sikic:** Formal analysis; Investigation; Writing – review & editing. **Markus Eckstein:** Formal analysis; Investigation; Writing – review & editing. **Arne Strauß:** Resources; Supervision; Writing – review & editing. **Friedemann Zengerling:** Resources; Supervision; Writing – review & editing. **Michael Hölzel:** Resources; Supervision; Writing – review & editing. **Philip Zeuschner:** Formal analysis; Investigation; Methodology; Project administration; Validation; Writing – original draft. **Charis Kalogirou:** Conceptualization; Investigation; Project administration; Resources; Supervision; Validation; Writing – review & editing.

## Ethics approval

This study was approved by the responsible ethical review board (20201211‐01).

## Data Availability

The data that support the findings of this study are available from the corresponding author upon reasonable request.
