# Supplementary material for: C‐reactive protein flare‐response predicts long‐term efficacy to first‐line anti‐PD‐1‐based combination therapy in metastatic renal cell carcinoma
Source: Clin Transl Immunology. 2021 Dec 6;10(12):e1358. doi: 10.1002/cti2.1358 (PMC8648498; doi:10.1002/cti2.1358)
Supplement: Supplementary file 1 [file CTI2-10-e1358-s001.docx]

**Supplementary table 1.** Uni- and multivariable Cox regression analysis for overall survival.

|  | **univariate** | | **multivariate** | |
| --- | --- | --- | --- | --- |
|  | **HR (95%CI)** | ***P*-value** | **HR (95%CI)** | ***P*-value** |
| **Therapy**  **IO+IO**  **IO+TKI** | ref.  0.57 (0.18; 1.80) | 0.334 | ref.  0.00 (0.00; 35203) | 0.147 |
| **CRP dynamics**  **no response**  **responder**  **flare-responder** | ref.  0.54 (0.17; 1.73)  0.25 (0.03; 2.01) | 0.301  0.301  0.193 | ref.  0.81 (0.00; 1.2e21)  0.00 (0.00; 1.6e77) | 0.980  0.993  0.945 |
| **Baseline CRP** | 1.04 (0.99; 1.08) | 0.055 | 1.59 (0.85; 3.00) | 0.149 |
| **Age** | 1.02 (0.98; 1.07) | 0.339 | 4.39 (0.68; 28.29) | 0.120 |
| **Gender**  **male**  **female** | ref.  1.45 (0.52; 4.10) | 0.481 | ref.  0.00 (0.00; 4.84e13) | 0.254 |
| **ECOG**  **0**  **1**  **2**  **3** | ref.  1.38e6 (0.00; 1.86e104)  1.15e7 (0.00; 1.55e105)  0.36 (0.00; 0.00) | 0.008  0.919  0.905  0.999 | ref.  3.03e54 (0.00; 2.32e136)  9.10e61 (0.00; 2.19e153)  1.70e84 (0.00; 0.00) | 0.621  0.192  0.184  0.934 |
| **IMDC**  **good**  **intermediate**  **poor** | ref.  4.18 (0.53; 33.01)  2.40 (0.22; 26.47) | 0.349  0.175  0.476 | ref.  6.06e15 (0.00; 1.06e66)  0.00 (0.00; 9.16e41) | 0.366  0.538  0.697 |
| **Histology**  **clear cell**  **non clear cell** | ref.  3.20 (0.93; 11.01) | 0.065 | ref.  0.00 (0.00; 8.58e25) | 0.692 |
| **pT stadium**  **pT1**  **pT2**  **pT3**  **pT4** | ref.  0.55 (0.07; 4.62)  0.70 (0.20; 2.50)  1.07 (0.13; 9.10) | 0.909  0.582  0.585  0.948 | ref.  0.053 (0.00; 22428.04)  0.000 (0.00; 1.91e17)  0.77 (0.00; 7866.34) | 0.904  0.657  0.487  0.956 |

**
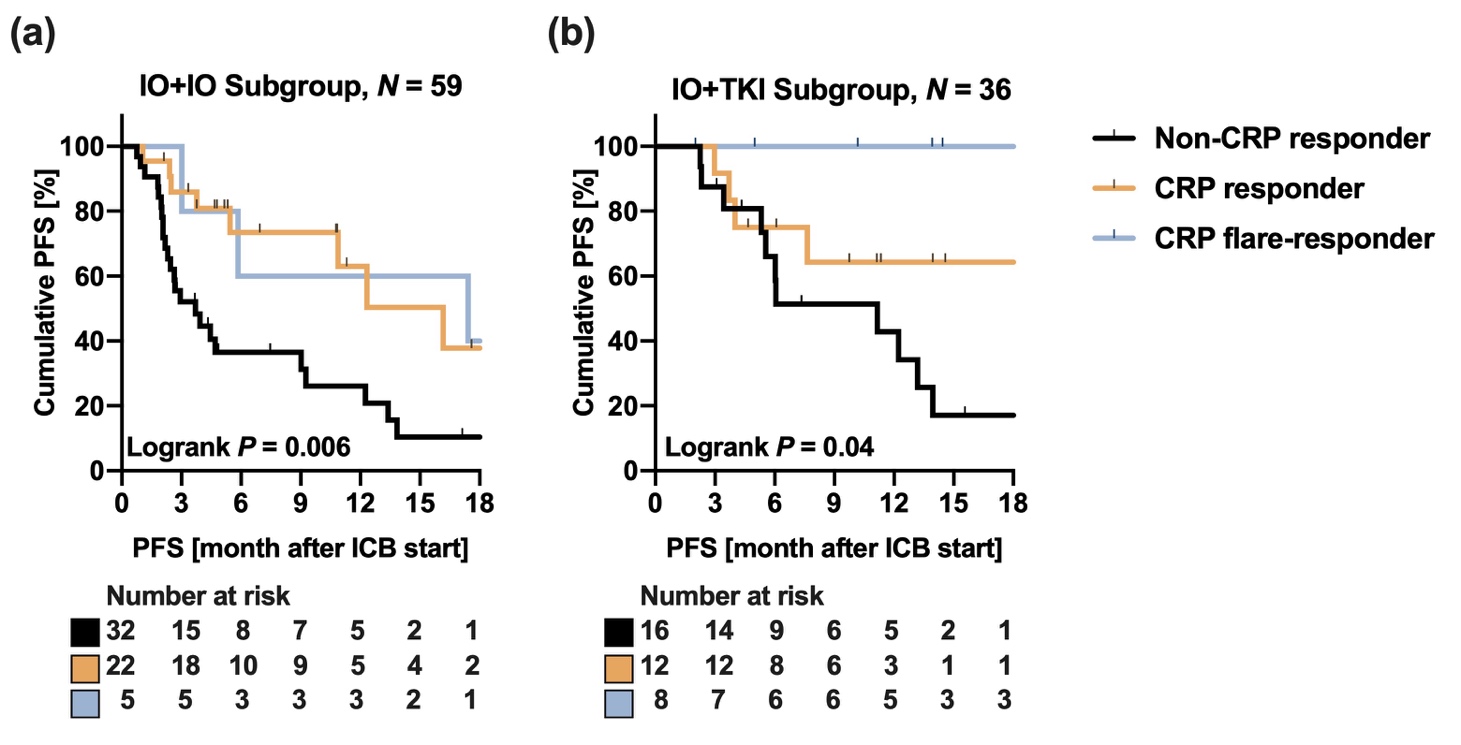
Supplementary figure 1.** Association of CRP kinetics subgroups and progression-free (PFS) survival after treatment initiation for the subgroup of patients receiving either first line **(a)** IO+IO (*N*=59) or **(b)** IO+TKI (*N*=36).
